# Indocyanine green (ICG) fluorescent cholangiography during laparoscopic cholecystectomy using RUBINA™ technology: preliminary experience in two pediatric surgery centers

**DOI:** 10.1007/s00464-021-08596-7

**Published:** 2021-07-06

**Authors:** Ciro Esposito, Daniele Alberti, Alessandro Settimi, Silvia Pecorelli, Giovanni Boroni, Beatrice Montanaro, Maria Escolino

**Affiliations:** 1 Division of Pediatric Surgery, Federico II University of Naples, Via Pansini 5, 80131 Naples, Italy; 2 Division of Pediatric Surgery, ASST Spedali Civili, Brescia, Italy

**Keywords:** Indocyanine green, Fluorescence, Cholangiography, Technology, Cholecystectomy, Children

## Abstract

**Background:**

Recently, we reported the feasibility of indocyanine green (ICG) near-infrared fluorescence (NIRF) imaging to identify extrahepatic biliary anatomy during laparoscopic cholecystectomy (LC) in pediatric patients. This paper aimed to describe the use of a new technology, RUBINA™, to perform intra-operative ICG fluorescent cholangiography (FC) in pediatric LC.

**Methods:**

During the last year, ICG-FC was performed during LC using the new technology RUBINA™ in two pediatric surgery units. The ICG dosage was 0.35 mg/Kg and the median timing of administration was 15.6 h prior to surgery. Patient baseline, intra-operative details, rate of biliary anatomy identification, utilization ease, and surgical outcomes were assessed.

**Results:**

Thirteen patients (11 girls), with median age at surgery of 12.9 years, underwent LC using the new RUBINA™ technology. Six patients (46.1%) had associated comorbidities and five (38.5%) were practicing drug therapy. Pre-operative workup included ultrasound (*n* = 13) and cholangio-MRI (*n* = 5), excluding biliary and/or vascular anatomical anomalies. One patient needed conversion to open surgery and was excluded from the study. The median operative time was 96.9 min (range 55–180). Technical failure of intra-operative ICG-NIRF visualization occurred in 2/12 patients (16.7%). In the other cases, ICG-NIRF allowed to identify biliary/vascular anatomic anomalies in 4/12 (33.3%), including Moynihan's hump of the right hepatic artery (*n* = 1), supravescicular bile duct (*n* = 1), and short cystic duct (*n* = 2). No allergic or adverse reactions to ICG, post-operative complications, or reoperations were reported.

**Conclusion:**

Our preliminary experience suggested that the new RUBINA™ technology was very effective to perform ICG-FC during LC in pediatric patients. The advantages of this technology include the possibility to overlay the ICG-NIRF data onto the standard white light image and provide surgeons a constant fluorescence imaging of the target anatomy to assess position of critical biliary structures or presence of anatomical anomalies and safely perform the operation.

**Supplementary Information:**

The online version contains supplementary material available at 10.1007/s00464-021-08596-7.

Despite widespread implementation of laparoscopic cholecystectomy (LC) in the pediatric population [1, 2], serious complications such as bile duct injury (BDI) and conversion to open continue to be ongoing occurrences [3, 4]. Though infrequent, BDI may cause significant patient morbidity as serious injuries often require complex reconstructive surgery, with variable long-term outcomes [5, 6].

The reported prevalence of BDI following LC ranges from 0 to 7% in early reports [7]. LC seems to be associated with a higher incidence of BDI than previous reports of open cholecystectomy, usually resulting from poor visualization/misinterpretation of anatomic structures [6–8]. Possible explanations include variant anatomy of the biliary tract, obesity, recurrent cholecystitis/cholangitis with resultant adhesions to surrounding structures, fibrosis and distorted anatomy plus failure to obtain an operative cholangiogram, inadequate dissection, injudicious use of cautery or clip placement, or the limited surgical experience [7, 9, 10]. Literature, however, continues to indicate the primary cause of BDI is an error in visual perception of the anatomy in 71–97% of cases [11].

This finding has prompted surgeons to search for reliable methods to avoid BDI while performing LC. The Critical View of Safety (CVS) has been described as a preventive measure to decrease the risk of injury to extrahepatic bile ducts [12]. The use of intra-operative cholangiography (IOC) has been recommended to help prevent misinterpretation of biliary anatomy, though its routine use is debated [6, 13]. More recently, indocyanine green fluorescent cholangiography (ICG-FC) has been adopted as a valid alternative to routine IOC for anatomic delineation of the extrahepatic biliary tract in the adult population [11, 14–16].

We reported the feasibility of ICG near-infrared fluorescence (NIRF) imaging to obtain detailed anatomical mapping of extrahepatic biliary structures during LC in the pediatric population as well [17–19]. We also standardized modality of applications of ICG-NIRF, such as dosage and timing of administration, in the pediatric patients [20].

This paper aimed to describe the use of a new technology, RUBINA™, to perform intra-operative ICG-FC during pediatric LC.

## Materials and methods

### Patient selection

Patients undergoing LC at two divisions of Pediatric Surgery from January 2020 to January 2021, received intraoperatively ICG-FC using the new technology RUBINA™. All the surgical procedures were performed by three senior surgeons in the two centers.

Inclusion criteria were patients < 18 years old with a history of symptomatic biliary disease (chronic cholecystitis, symptomatic cholelithiasis) as well as acute cholecystitis and cholangitis.

Exclusion criteria included patients who were pregnant or those allergic to iodide or shellfish or those with inadequate documentation regarding ICG-FC use or those needing conversion to open surgery.

The study was approved by the appropriate Institutional Review Board (IRB) at each institution.

### Operative technique

Patients were admitted the day before surgery to give the ICG injection. A vial of ICG (25 mg) was diluted with 10 mL of sterile water. Once reconstituted, the ICG solution was injected via the intravenous route using a dosage of 0.35 mg/Kg. The median timing of administration was 15.6 h prior to surgery (range 8–20). Following the administration, patients were monitored for any signs of allergic reaction to the dye.

**Fig. 1:**
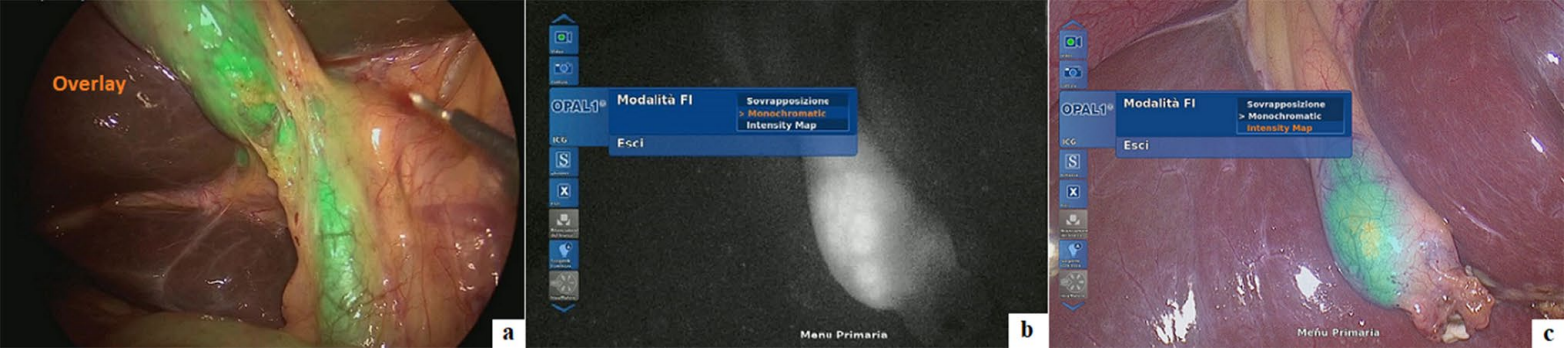
ICG-NIRF view modes using RUBINA™: overlay (**a**), monochromatic (**b**), and intensity map (**c**)

The surgical procedure was performed using IMAGE 1 S™ RUBINA™ system, manufactured by KARL STORZ SE & CO. KG, Tuttlingen, Germany. This system is composed of OPAL1® NIR/ICG, a camera head for ICG-NIRF imaging in combination with POWER LED RUBINA™ and TIPCAM®1 RUBINA™, a high-resolution laparoscope with two distally integrated video chips for ICG-NIRF imaging, direction of view 30°, and diameter of 10 mm. A four-trocar laparoscopic cholecystectomy was performed in all cases. After trocar placement and induction of pneumoperitoneum, the ICG-NIRF was activated by pushing a button on the camera head and allowed real-time fluorescent visualization of extrahepatic biliary structures before dissection of the Calot’s triangle. The RUBINA™ components offer various new modes for visualizing the ICG-NIRF signal. In overlay mode, the ICG-NIRF data are overlapped onto the standard white light image to generate an overlay image (Fig. 1a). Depending on your preferences and application, the ICG-NIRF data can be displayed as a green or blue overlay. In the monochromatic mode, the ICG-NIRF signal alone is displayed in white on a black background to achieve the greatest possible differentiation (Fig. 1b). Finally, the intensity map mode displays the intensity of the ICG-NIRF signal using a color scale in an overlay image (Fig. 1c). The specific view mode can be selected in the menu on the camera head. We preferentially adopted the overlay mode to perform ICG-FC in all cases. In fact, using the overlay of ICG-NIRF signal onto the standard white light image provided by RUBINA™, we adopted NIRF imaging throughout the entire operation during the dissection, without the need to switch between standard camera views to those with ICG-NIRF to more definitively identify the biliary tree anatomy, as happened using the existing IMAGE1 S™ camera platform.

**Fig. 2:**
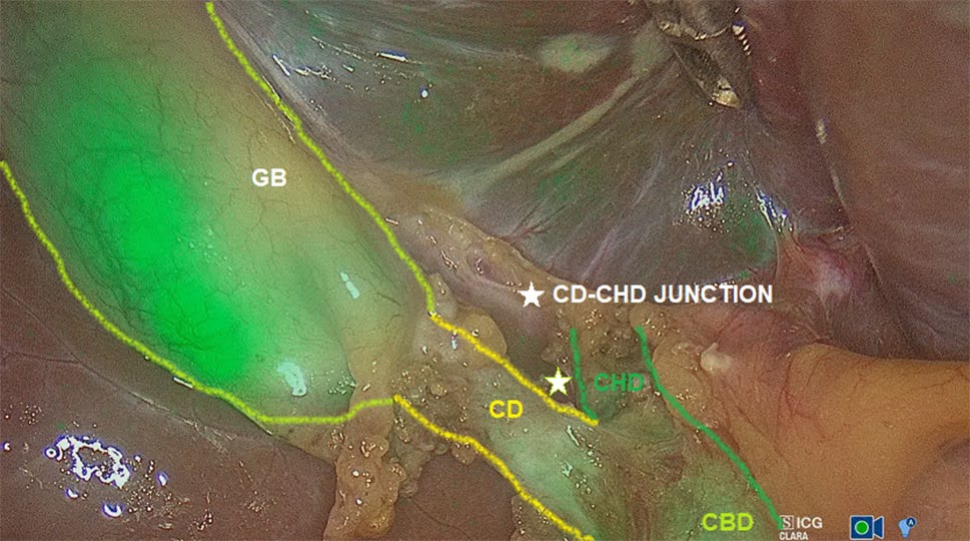
The gallbladder (GB), the cystic duct (CD), the common hepatic duct (CHD), the CD–CHD junction, and the common biliary duct (CBD) were easily detected using ICG-FC

**Fig. 3:**
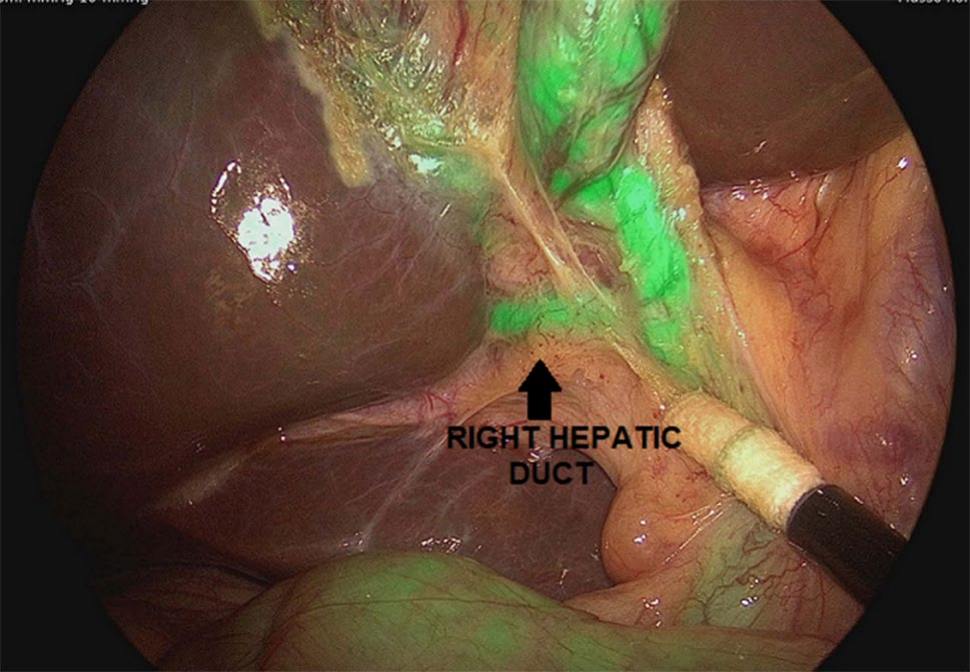
Right hepatic duct (arrow) was visualized using ICG-FC

LC was performed by obtaining the critical view of safety (CVS) in all cases. Use of ICG-FC was very helpful to easily detect the cystic duct (CD), the common hepatic duct (CHD), the CD–CHD junction, the common bile duct (CBD), the right hepatic duct, and accessory hepatic ducts and did not require any additional ports or instrumentation (Figs. 2 and 3). The CD was clipped using 5-mm clip appliers with two clips proximal from CBD take off and one to two clips distally. The cystic artery was slowly cauterized using hook electrocautery or clipped and divided. The gallbladder was then removed from the gallbladder fossa with hook electrocautery and extracted through the umbilical trocar’s orifice. The liver bed was then examined for hemostasis.

The ICG-FC view was also helpful during the removal of the gallbladder allowing to identify the dissection plane between the gallbladder and undersurface of the liver. Finally, the anatomy was reidentified, confirming that the CBD was uninjured. In most cases, a drain was routinely placed in the hepatic fossa for at least 12 h postoperatively, according to the surgeon’s personal preference. Trocars’ orifices were closed using resorbable stitches.

The steps of the operative technique are shown in Video 1.

### Data collection

Patient baseline, intra-operative details, rate of biliary anatomy identification, utilization ease, and surgical outcomes were assessed.

## Results

Thirteen patients (11 girls and 2 boys), with median age at surgery of 12.9 years (range 6.5–18), underwent LC and received ICG-FC using the new RUBINA™ technology. The median body mass index (BMI) was 22.2 kg/m^2^ (range 12.4–34.8). Based upon their BMI, 2 patients were underweight (< 18.5 kg/m^2^), 7 patients were ideal weight (≥ 18.5–24.9 kg/m^2^), 1 was overweight (≥ 25–29.9 kg/m^2^), and 3 were obese (> 30 kg/m^2^). Indications for surgery included symptomatic cholelithiasis/biliary colic (*n* = 9), prior acute cholecystitis treated nonoperatively (n = 3), and chronic cholecystitis (n = 1). Six patients (46.1%) had associated comorbidities including drepanocytosis (*n* = 2), epilepsy (*n* = 1), chronic hepatitis B (*n* = 1), hereditary spherocytosis (*n* = 1), Mirizzi syndrome (*n* = 1), and Crigler–Najjar syndrome type II (*n* = 1). Five patients (38.5%) were practicing drug therapy at time of surgery including phenobarbital (*n* = 1), sodium valproate (*n* = 1), and ursodeoxycholic acid (UDCA) (*n* = 5). Pre-operative workup included ultrasound (*n* = 13) and cholangio-MRI (*n* = 5), excluding biliary and/or vascular anatomical anomalies.

Patient baseline is reported in Table 1.

**Table 1:** Patient baseline

Patient *n*	Gender	Age at surgery (years)	Weight (Kg)	BMI (Kg/m2)	Comorbidity	Drug therapy at time of surgery	Pre-operative Ultrasound	Pre-operative MRI	Pre-operative diagnosis anatomy variants	Timing administration ICG prior to surgery (hours)
1	F	13.1	25	12.4	Drepanocytosis, epilepsy	Sodium valproate, ursodeoxycholic acid (UDCA)	Yes	Yes	No	8
2	F	7.4	26	16.9	Hereditary spherocytosis	No	Yes	Yes	No	13
3	F	15.7	45	19.2	No	UDCA	Yes	Yes	No	12
4	F	12.9	51	19.6	Drepanocytosis	Amoxicillin	Yes	Yes	No	13
5	F	6.4	32	21.9	Mirizzi’s syndrome	UDCA	Yes	Yes	No	14
6	F	12	71	27.7	No	No	Yes	No	No	21
7	F	18	87	30.1	No	No	Yes	No	No	20
8	F	13	61.2	24.5	No	No	Yes	No	No	18
9	M	18	65.5	23.2	No	No	Yes	No	No	20
10	F	13	87	34.8	No	No	Yes	No	No	18
11	M	17	88.5	31.3	Chronic hepatitis B	No	Yes	No	No	20
12	F	17	56	21.3	Crigler–Najjar syndrome type II	Phenobarbital	Yes	No	No	18
13	F	9	37.5	19.1	No	No	Yes	No	No	19

**Fig. 4:**
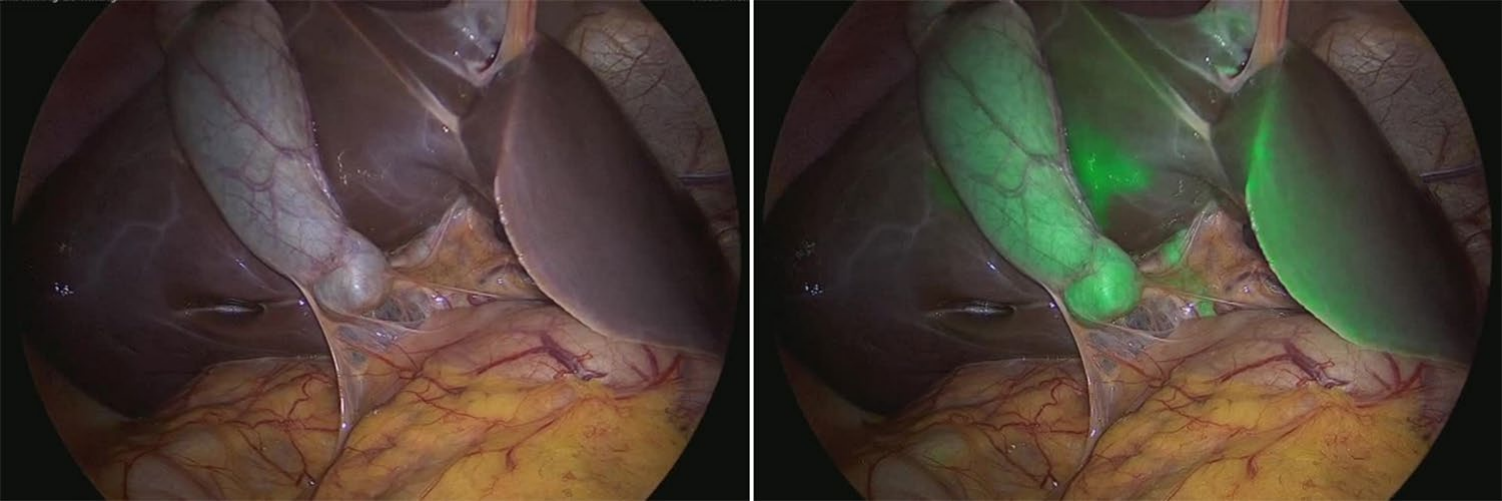
Hyperfluorescence of liver background following early administration of ICG (8 h prior to surgery)

**Fig. 5:**
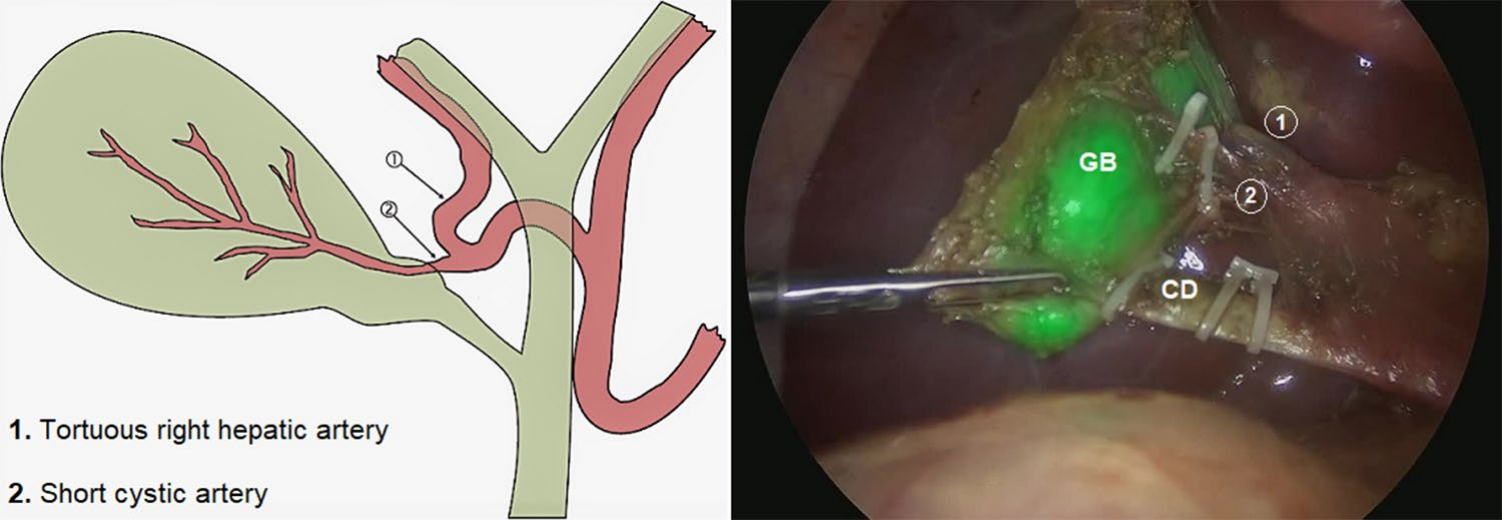
Caterpillar or Moynihan’s hump: a tortuous right hepatic artery (1) and a short cystic artery (2) coming very close to the gallbladder (GB) and cystic duct (CD)

**Fig. 6:**
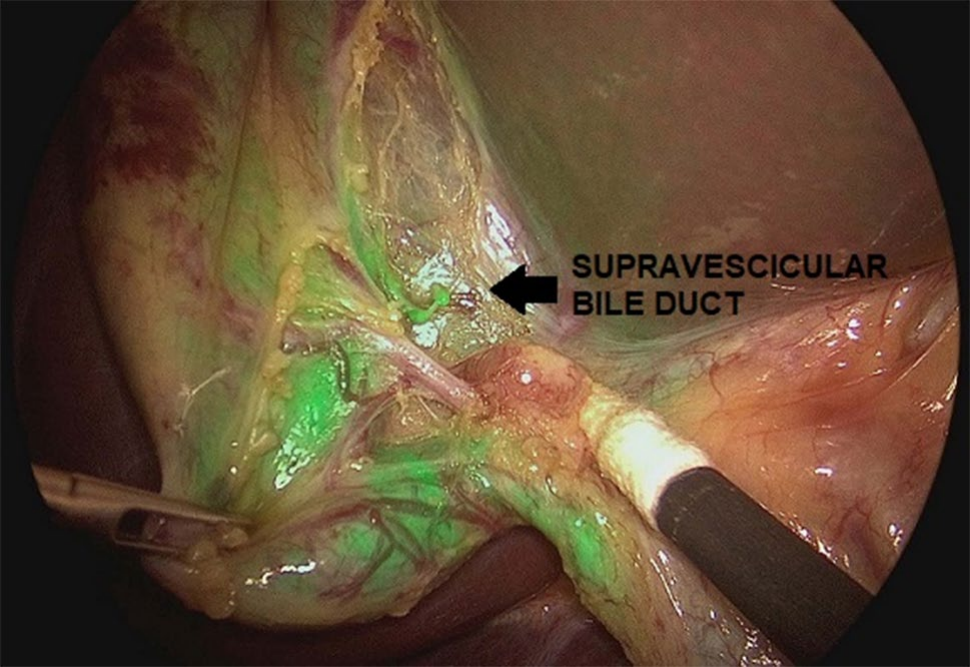
Supravescicular bile duct (arrow) was discovered intraoperatively using ICG-FC

One patient needed conversion to open surgery due to technical challenges and was excluded from the study. The median operative time was 96.9 min (range 55–180). Technical failure of intra-operative ICG-NIRF visualization occurred in 2/12 patients (16.7%), including absence of fluorescence (*n* = 1) and hyperfluorescence of liver background (*n* = 1) (Fig. 4). In the other cases, ICG-enhanced fluorescence imaging allowed to identify biliary/vascular anatomic anomalies in 4/12 (33.3%), including Moynihan or caterpillar hump of the right hepatic artery (*n* = 1), that is a tortuous right hepatic artery producing sinuosity and coming very close to the gallbladder and cystic duct (Fig. 5), supravescicular bile duct (*n* = 1) (Fig. 6), and short cystic duct (*n* = 2).

No allergic or adverse reactions to ICG injection, post-operative complications, or reoperations were reported.

Patient outcomes are reported in Table 2.

**Table 2:** Patient outcomes

Patient *n*	Operative time (minutes)	Intra-operative complications	Technical problems of ICG-NIRF	Intra-operative diagnosis of variant anatomy	Post-operative complications	Allergy and/or reactions to ICG	Conversion to open surgery	Reoperation
1	95	No	Hyperfluorescence liver background	No	No	No	No	No
2	145	No	No	Moynihan’s hump	No	No	No	No
3	180	No	No	No	No	No	No	No
4	100	No	No	Accessory hepato-cystic duct	No	No	No	No
5	420	No	No	No	No	No	Yes	No
6	55	No	No	No	No	No	No	No
7	78	No	No	No	No	No	No	No
8	115	No	No	Gallbladder indented in the liver fossa	No	No	No	No
9	85	No	No	Short and angled cystic duct	No	No	No	No
10	62	No	No	No	No	No	No	No
11	58	No	No	No	No	No	No	No
12	125	No	Absent fluorescence	No	No	No	No	No
13	65	No	No	Short cystic duct	No	No	No	No

## Discussion

The primary goal of any surgery is to achieve optimal surgical results. To meet this objective, the visualization and display of significant and critical structures are of crucial importance to the surgical workflow. The rapid development of technology has led to greater insights in the field of minimally invasive surgery (MIS) and, ultimately, to a potentially better outcome for the patient.

As in adults, laparoscopic cholecystectomy (LC) has now become the technique of choice also in the pediatric population [3]. However, despite the improvement in the learning curves of pediatric surgeons, the procedure is not without complications [3]. The most serious complications include biliary tree injuries, usually resulting from poor visualization/misinterpretation of anatomic structures [6–8].

As recently published [17–20], the incorporation of ICG fluorescent cholangiography (FC) into LC has the potential to provide real-time visualization of the extrahepatic biliary tree prior to commencing dissection within the Calot’s triangle. The evolving technologies have recently provided the surgeons perfect camera systems to achieve even better intra-operative visualization.

The new IMAGE1 S™ RUBINA™ components provide surgeons a series of advantages compared with the existing IMAGE1 S™ camera platform. These options include native 4 K resolution, offering very good image quality in both white light and ICG-NIRF modes, and natural color rendition; possibility to adopt 3D technology in 4 K and enhanced 3D quality image compared to previous model; automatic horizon control; laser-free LED light source for white light, and ICG-NIRF providing excitation of ICG and autofluorescence in the near-infrared range, durability and constant light intensity, and control via touch display and footswitch. The OPAL1® ICG-NIRF technology integrated into the RUBINA™ camera platform provides the overlay mode with ICG-NIRF data displayed in green or blue onto the standard white light image or intensity map mode for displaying signal intensity in the overlay image or monochromatic mode for ICG-NIRF signal alone (Fig. 1). In our experience, we preferentially adopted the overlay mode, as the ICG-NIRF data overlapped onto the standard white light image allowed us to keep a constant assessment of anatomy and continuously identify the position of critical biliary structures, while performing the operation without the need to switch between the ICG-NIRF and the standard bright light view.

Use of ICG-FC was very helpful to easily detect the cystic duct (CD), the common hepatic duct (CHD), the CD–CHD junction, the common bile duct (CBD), the right hepatic duct, and accessory hepatic ducts and did not require any additional ports or instrumentation (Figs. 2 and 3). Improved anatomical identification allowed for targeted dissection of the CD and aided to confirm cystic artery versus CD during dissection. We found the use of ICG-FC very useful to orient the surgeon to the location of biliary structures, particularly in the setting of excess adipose tissue overlying the Calot’s triangle, or adhesions resulting from inflammatory processes, or in case of aberrant anatomy.

When performing cholecystectomy, the surgeon must remember that there are many variations from the normal anatomy of the vessels and bile ducts in the Calot’s triangle. Thanks to the use of ICG-FC, we were able to identify anatomy variants in 33.3% of our cases, including Moynihan’s or caterpillar hump of the right hepatic artery, supravescicular bile duct, and short cystic duct.

Numerous variations in origin and branching pattern of right hepatic artery have been reported. In some cases, tortuous right hepatic artery producing sinuosity may come very close to the gallbladder and cystic duct in the form of “caterpillar hump or Moynihan’s hump’’ [21]. If such a hump is present, the cystic artery in turn is very short (Fig. 5). In this situation right hepatic artery is either liable to be mistakenly identified as cystic artery or torn in attempts to ligate the cystic artery [22]. Injury to right hepatic artery leads to ischemic necrosis of right functional lobe of the liver. So, the presence of caterpillar hump should be suspected when an unusually large cystic artery is viewed through the laparoscope [23].

Another debated point is the optimal dosage and timing of administration of ICG to obtain optimal visualization of the extrahepatic biliary tree [24, 25]. We adopted a dosage of 0.35 mg/kg in all cases, and the median time of administration was 15.6 h prior to surgery. Strong fluorescence of the background tissues was visualized intraoperatively in one patient, in whom the ICG administration was performed just 8 h prior to the procedure (Fig. 4). Based upon our experience, we believe that ICG administration should be performed the day before surgery, average 16–18 h prior to the procedure, to obtain better fluorescence contrast between the bile duct and background tissues, thereby enhancing the efficacy of ICG-FC during LC.

It is also important to consider that some drugs can interfere with the ICG mechanism of action. In our series, we reported a technical failure in intra-operative visualization of ICG-NIRF in one patient affected by Crigler–Najjar syndrome type 2, who was in treatment with phenobarbital. In such patient, we observed a weak fluorescence of biliary structures, which did not improve following intra-operative administration of additional ICG solution.

This study has further confirmed that ICG-FC during LC has the potential to increase both patient safety and procedural efficiency via enhancement and optimization of tissue visualization. No post-operative complications occurred in our series. However, conversion from laparoscopic to open cholecystectomy is sometimes mandatory, due to inability to identify anatomy, need to avoid injury, or insurance of patient safety. Furthermore, this technology is very easy to use as ICG-NIRF view can be directly activated by pushing a button on the camera without increased need for staffing or additional supplies in the operative theater, beyond the addition of a NIR-capable laparoscopic equipment.

Regarding the costs to adopt this innovative imaging technology, you need to buy the IMAGE1 S ™ RUBINA™ system, including the specific camera head and the light source, and a separate high-resolution scope, TIPCAM®1 RUBINA™, with two distally integrated video chips for ICG-NIRF imaging, manufactured by KARL STORZ SE & CO. KG, Tuttlingen, Germany. The cost of the IMAGE1 S™ RUBINA™ system is about 50.000 Eur, whereas the cost of the scope, TIPCAM®1 RUBINA™, is about 5.000 Eur. The ICG dye costs about 40 Eur per vial. So, considering that this technology is easy to use, safe, and versatile, if the operating room is provided with all the equipment needed for ICG-NIRF, it may be adopted in every routine pediatric LC with practically no adjunctive costs except for the ICG vial.

No absolute contraindications exist for the administration of ICG dye, and ICG has been used safely in patients with documented iodine allergy [26, 27]. Risk of an adverse event with ICG is small. Anaphylactic reaction has been reported at a rate of 0.003%, with a 0.34% overall incidence of mild adverse reactions [26, 27]. We adhere to the general recommendation that ICG not to be used for patients with a shellfish allergy or iodine contrast sensitivity, and we did not experience any adverse reactions with the use of ICG.

Our preliminary experience suggested that the new RUBINA™ technology was very effective to perform ICG-FC during LC in pediatric patients. The advantages of this technology include the possibility to overlay the ICG-NIRF data onto the standard white light image and provide surgeons a constant fluorescence imaging of the target anatomy to assess position of critical biliary structures or presence of anatomical anomalies and safely perform the operation.

## Supplementary Information

Below is the link to the electronic supplementary material.

Supplementary file1 (MP4 272164 kb)
